# Kommerell’s Diverticulum in a Right-Sided Aortic Arch With an Aberrant Left Subclavian Artery Hybrid Repair

**DOI:** 10.1177/15385744231183310

**Published:** 2023-06-13

**Authors:** Antonio Solano, Alejandro Pizano, Jawaher Azam, Gerardo Gonzalez-Guardiola, Michael Siah, Khalil Chamseddin, Vivek Prakash, Melissa L. Kirkwood, Michael Shih

**Affiliations:** 1 Division of Vascular and Endovascular Surgery, Department of Surgery, University of Texas, Dallas, TX, USA; 2 Medical School, 25989 University of Texas Southwestern Medical Center, Dallas, TX, USA Presented at the poster session of the Critical Issues America Annual Meeting. Miami, FL, February 10-11, 2023

**Keywords:** aortic arch anomalies, aortic arch aneurysm, aberrant subclavian artery, hybrid repair, Kommerell’s diverticulum, right aortic arch, thoracic endovascular aortic repair, total debranching

## Abstract

**Background:**

Kommerell’s diverticulum (KD) with a right aortic arch (RAA) and aberrant left subclavian artery (aLSCA) is a rare congenital anomaly of the aortic arch. Treatment is not well defined due to its uncommon presentation, with rupture and dissection risk rates of up to 53%.

**Case summary:**

A 54-year-old male with a history of chronic obstructive pulmonary disease (COPD) and hypertension presented with difficulty breathing during exercise without dysphagia. Follow-up computerized tomography angiogram (CTA) revealed the presence of a RAA and aLSCA arising from the descending thoracic aorta with an adjacent 58 × 41-mm KD and tracheal and esophageal displacement. Due to the size of the KD, risk of rupture, unsuitable anatomy for total endovascular aortic repair (EVAR), and high COPD burden, the patient was planned to undergo a hybrid surgical repair. Left common carotid (LCCA) artery to LSCA bypass, full aortic debranching, LSCA embolization and percutaneous thoracic endovascular aortic repair (TEVAR) were performed. Successful device position and exclusion of the diverticulum and aneurysmal aorta were observed after completion thoracic aortogram. 18-month follow-up CTA demonstrated patency of the LSCA to LCCA bypass graft and arch vessel branches, as well as stable exclusion of the KD. Persistence of a type II endoleak originated at the right first posterior intercostal artery has been noted and is being followed conservatively since no sac growth has occurred.

**Conclusion:**

We highlight the presence of a KD with RAA and aberrant subclavian artery, a rare congenital anatomic variation of the aortic arch with complex anatomy. Surgical planning must be individualized according to comorbidities and anatomical variations identified on imaging and 3D reconstructions.

## Background

Kommerell’s diverticulum (KD) is an uncommon developmental abnormality of the aorta consisting of a congenital abnormality of the aortic arch due to failed regression of the fourth primitive dorsal arch.^1^ This condition is associated with a left aortic arch (LAA) with an aberrant right subclavian artery (aRSCA) or a right aortic arch (RAA) with an aberrant left subclavian artery (aLSCA) in .5%- to 2.0% and .05% to .1% of the population, respectively.^1,2^ The literature has reported less than 50 cases of KD, which usually are asymptomatic and incidentally discovered in radiologic studies for other reasons.^3,4^ Treating KD with RAA and aLSCA is not well defined due to uncommon presentation, even though rupture or dissection risk rates in long-term follow-up studies ascend to 53%.^5^ A surgical approach is considered for both symptomatic and asymptomatic cases to reduce rupture and mortality rates. Detailed preoperative comprehension of the anatomy of the aortic arch and branches is needed for surgical planning, reducing complications, and improving long-term outcomes. 3D imaging reconstruction eases the preoperative identification of difficult anatomic variations. Nonetheless, standardizing open repair vs endovascular repair has not been established.^6,7^ We report a case of KD with RAA and aLSCA treated with a hybrid surgical approach. Verbal consent was obtained from the patient for publication of this case report.

## Case Report

A 54-year-old male with a history of chronic obstructive pulmonary disease (COPD) and hypertension presented with difficulty breathing during exercise (shortness of breath after walking for 1 mile and 3 flights of stairs) without dysphagia. Physical examination showed full symmetric palpable femoral and pedal pulses, with the rest of the findings reported as unremarkable.

**Figure 1.**
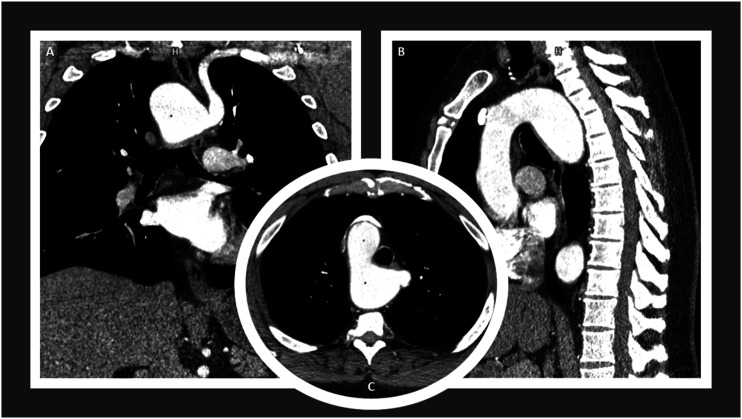
Preoperative computed tomography angiogram findings. Coronal view (A), sagittal view (B), and axial view (C) demonstrate a stable appearance of the right-sided aortic arch which crosses posterior to the esophagus with aneurysmal dilatation due to diverticulum of Kommerell adjacent to an aberrant left subclavian artery measuring maximum angle corrected diameter of 58 × 41-mm at the origin of the aberrant left subclavian artery. The origin of the left subclavian artery crosses posterior to the trachea and esophagus. The diverticulum of Kommerell causes anterior displacement of the trachea. There is a leftward tracheal deviation from the right-sided aortic arch, with mild narrowing of the trachea at the level of the arch. The trachea at the level of the aortic arch measures 11 mm in diameter, versus 14 mm above, for example. There is also mild posterior compression on the trachea by the posterior aortic arch at the takeoff of the left subclavian artery. There is leftward and anterior displacement of the esophagus by the posterior aortic arch diverticulum.

**Figure 2.**
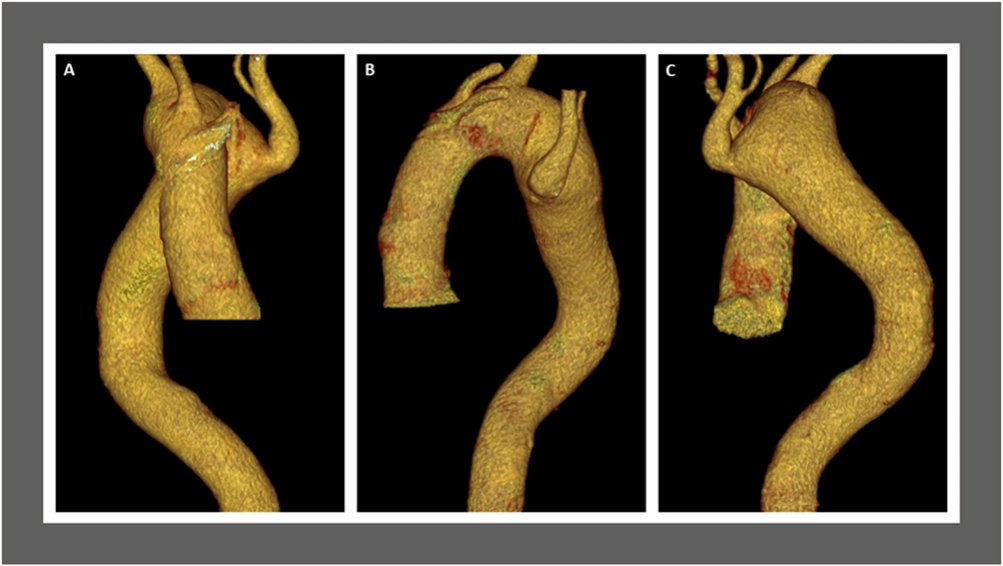
Preoperative computed tomography angiogram findings. Anterior view (A), left lateral view (B), and posterior view (B) of three-dimensional reconstruction with volume rendering technique demonstrate the arch vessels, ascending and descending aorta with aneurysmal dilatation due to diverticulum of Kommerell adjacent to an aberrant left subclavian artery at the origin of the aberrant left subclavian artery. The branches from the anterior aspect of the right-sided aortic arch are the left internal carotid artery, the right internal carotid artery, and the right subclavian artery. The aortic arch measures 3.7 cm in diameter. The distal aortic arch measures 5.2 cm in maximum transverse diameter. The diverticulum of Kommerell measuring maximum angle corrected diameter of 58 × 41-mm at the origin of the aberrant left subclavian artery.

During follow-up for the patient’s lung disease, a computed tomography angiogram (CTA) revealed the presence of an RAA and aLSCA arising from the descending thoracic aorta with an adjacent 58 × 41-mm KD and tracheal and esophageal displacement (Figures 1-2). Considering the size of the KD, the risk of rupture, the anatomy, and the high perioperative risk for traditional open surgery given his severe COPD, we recommended a hybrid surgical repair. Due to a lack of appropriate proximal landing zone, a complete arch debranching was necessary.

**Figure 3.**
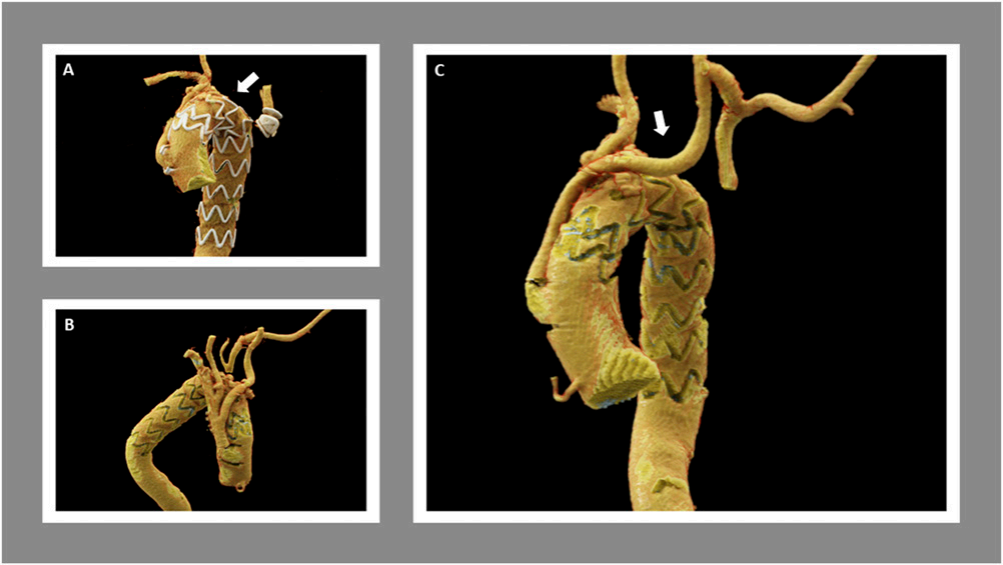
Six months follow-up computed tomography angiogram findings. Left anterolateral view (A), right lateral view (B), and left anterolateral view (C) of the three-dimensional cinematic volume rendering technique demonstrates right-sided aortic arch status post endovascular repair with reimplantation of common trunk supplying the innominate and left common carotid arteries (widely patent). The left common carotid to left axillary bypass graft is widely patent and exclusion the left subclavian artery at the origin with Amplatz plug. The artery distal to this is widely patent. The remaining portions of the visualized great vessels are widely patent. Type II endoleak arising from the first right posterior intercostal artery (arrows).

The left common carotid (LCCA) and left subclavian (LSCA) arteries were exposed initially with a horizontal supraclavicular incision, followed by median sternotomy and supra-aortic dissection. After heparinization, an 8-mm ring polytetrafluoroethylene graft was anastomosed to the LCCA to first be used as part of a cardiopulmonary bypass (CPB) circuit for anterograde cerebral perfusion. Then, an ascending aorta to innominate and LCCA bypass was performed, followed by cannulation of both the distal ascending aorta and LCCA bypass for CPB circuit formation. Given the abnormal quality of the aortic tissue and inability to oversew the origin of the great vessels with systolic flow through it, debranching of the arch vessels was performed under circulatory arrest for 7 minutes with adequate antegrade cerebral perfusion preservation. An 8-mm × 12-mm × 30 cm Hemashield trifurcation graft was selected and beveled to the appropriate length to perform end-to-end anastomosis with the arch vessels. The right subclavian artery was anastomosed to the 12-mm limb, followed by anastomosis of the LCCA to one of the 8-mm limbs and the right common carotid artery to the remaining 8-mm limb. Once the anastomoses were completed, a side-biting clamp was applied to the proximal segment of the ascending aorta. Then, an opening was created to anastomose the 12-mm stem of the trifurcation graft to the ascending aorta and rewarming was completed at this point Once no longer needed, the LCCA bypass that was used for the CPB was disconnected from the circuit and anastomosed to the LSCA to complete revascularization of the LSCA as a carotid-subclavian bypass. Following this, embolization of the proximal LSCA origin was performed using an 18-mm Amplatzer plug for the total exclusion of the KD. Finally, a 34 × 209-mm Cook Zenith Alpha endograft was deployed at the origin of the stem of the trifurcation graft covering the distal ascending aorta in Zone 0 (Figure 3). The completion thoracic aortogram confirmed the successful position of the device and the exclusion of the diverticulum and aneurysmal aorta. Wide patency of bypasses to all vessels (patent bilateral carotid arteries and left vertebral, right vertebral covered purposely by the thoracic endovascular aortic repair (TEVAR)) was observed. The patient tolerated the procedure well and was extubated with a normal neurologic exam and palpable distal pedal and radial pulses bilaterally.

**Figure 4.**
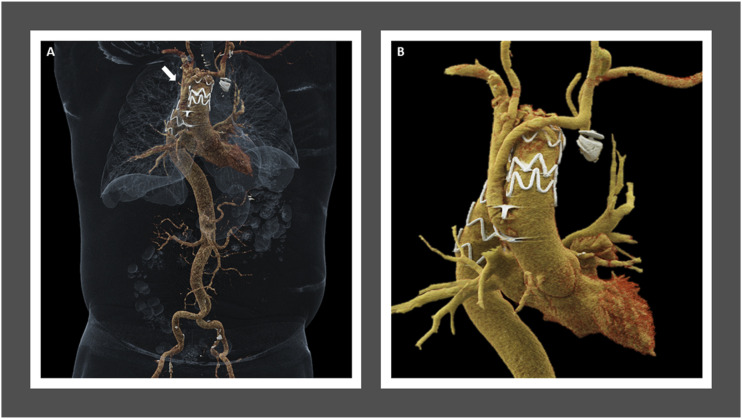
One-year follow-up computed tomography angiogram. Three-dimensional cinematic volume rendering technique anterior thoracoabdominal view (A) ascending and descending thoracic view (B) show right-sided aortic arch and post debranching the great vessels with reimplantation of the proximal aorta, left carotid to subclavian bypass with embolization of the proximal left subclavian artery and endovascular stent graft repair of thoracic aortic aneurysm. Reimplanted great vessels are all widely patent. Endovascular stent graft extends from the mid-ascending aorta to the mid-descending aorta. Redemonstration of type II endoleak (arrow). The distal aortic arch measures 50 mm in the axial plane at the level of the Amplatzer occlusion plug of the left subclavian artery, which is unchanged when remeasured by me at the same level in the prior study.

Postoperatively, the patient developed an *E. coli* urinary tract infection and bacteremia which were treated with intravenous antibiotic therapy. Otherwise, the hospital course was uncomplicated, and he was discharged on postoperative day 11. At 30-day follow-up visit, his symptoms of dyspnea had significantly improved. Computed tomographic angiography demonstrated a patent ascending aorta to innominate and LCCA bypass as well as a patent LCCA to LSCA bypass graft with evidence of a small type II endoleak arising from the right first posterior intercostal artery. 3-, 6-, 12-, and 18-months follow-up CTAs have demonstrated a stable excluded aneurysm sac at the aortic arch as well as the persistence of the small type II endoleak (Figure 3-4) that is being followed conservatively due to continued sac shrinkage.

## Discussion

The literature has reported 32 documented cases like this patient, arising KD with RAA and aLSCA as an uncommon clinical presentation.^5^ The etiology of RAA anomalies is currently unknown and has been linked to a deletion in chromosome 22q11 to RAA anomalies with an incidence of 24% of isolated anomalies of laterality of branching of the aortic arch.^8^ Suspicion of this clinical condition can arise in patients with a previous history of congenital heart anomalies or compression of mediastinal structures such as the trachea or the esophagus during childhood, and adults who experience symptoms secondary to compression of surrounding structures including dysphagia, respiratory symptoms (dyspnea, stridor, wheezing, cough, choking spells, recurrent pneumonia, obstructive emphysema), or chest pain.^9^

Kommerell’s diverticulum is a rare condition that can occur in cases of RAA with aLSCA, leading to saccular aneurysmal dilatation and subsequent mortality. Surgical indication has not been established due to the uncommon presentation of this condition. Still, it may be considered for symptomatic KD and dimensions > 50 mm in the long-axis or > 30 mm for orifice diameter.^1,10^ Likewise, an aggressive intervention strategy is suggested based on rupture rates up to 53%.^5^ Spontaneous rupture may occur independently from the diverticulum dimension due to increased shear stress exerted by RAA, as well as the potential contribution of primary vascular pathology or premature atherosclerosis.^11^ Since our patient was symptomatic and the aneurysm sac size was 58 mm, surgery was advised.

We highlight the atypical anatomical variation challenge for the procedure as well as the scarce number of hybrid repairs reported in the literature for this specific clinical vignette. Gray et al. evaluated the safety and efficacy of hybrid approach for repair of complicated aberrant subclavian arteries. Out of 18 patients, only 1 case of KD with RAA and aLSCA was reported. 1-year and 3-year-survival rates were 93% ± 6% and 84% ± 10%, respectively, and only 3/8 patients who presented endoleaks required intervention during follow-up.^12^ In light of these results, hybrid procedures have an adequate safety profile. It is imperative to discuss with patients surveillance follow-up imaging and the need of reinterventions as adjunctive measures to ensure procedure long-term durability. Despite not being reported as the first case of hybrid repair for KD with RAA and aLSCA, this case can further support this approach as an alternative for scenarios of complex anatomical variations.

Three types of aortic arch diverticulum have been described: diverticulum in a LAA with aRSCA, diverticulum in a RAA with aLSCA, and diverticulum at the aortic-ductal junction. Based on Edward’s classification,^13^ RAAs can also be subdivided. Type I includes RAA with mirror image arch branches. Type II includes RAA with aLSCA and KD, as described in this case. Type III includes RAA with isolated LSCA communicating with the pulmonary artery. Significant anatomical differences can be observed when comparing the aortic diverticulum in a LAA with aRSCA vs RAA with aLSCA. Up to 60% of aRSCA present from incomplete regression of the primitive distal RAA.^14^ The aRSCA arises from the descending aorta distal to the LSCA, crossing the midline posterior to the esophagus and producing a large oblique esophageal indentation. Conversely, the RAA diverticulum is usually a large rounded outpouching at the origin of the aberrant vessel, resulting from an interruption of the normal left arch between the LCCA and LSCA, and located lateral and posterior to the trachea and esophagus.

Different surgical techniques can be employed to treat KD. Currently, alternatives have expanded to include endovascular and hybrid repair for complex aortic disease.^5,10^ CTA with 3D reconstruction is the gold standard for establishing precise anatomy, identifying anatomic variations, and surgical planning. Open repair has been the mainstay of treatment to reconstruct aberrant subclavian artery anatomy^15^ and should be considered for symptomatic patients or aneurysm diameter >30 mm in the absence of symptoms.^16^ Good long-term outcomes are described with this approach.^17,18^ Associated drawbacks include high morbidity and mortality rates and technical anatomic difficulty. Our patient was not thought to be a good open thoracotomy candidate for total open repair due to his severe COPD. To perform endovascular repair, sufficient size of the access arteries, limited tortuosity of the aorta and aberrant vessel, and suitable proximal and distal neck morphology must be present.^19^ A sufficient proximal landing zone and absence of aberrant vessel branches are also needed to avoid type I and II endoleaks.^20^ Evidence regarding hybrid repair has reported decreased in-hospital mortality and shorter length of stay, posing this approach as a safe and effective treatment.^8,21^ Selection of the specific type of intervention must be based on patient anatomy, comorbidities, and surgeon’s experience.^9^

## Conclusions

Kommerell’s diverticulum in a right-sided aortic arch with an aLSCA is a rare congenital pathology. Diagnosis may be challenging due to its asymptomatic presentation. A timely diagnosis and referral to vascular surgery contribute to mortality and rupture risk reduction. Imaging and 3D reconstructions are essential for identifying anatomic vascular variations and surgical planning. Hybrid repair is a feasible and safe approach for aberrant vessels and aneurysm repair, which can support further use in this specific clinical scenario. Long-term surveillance is essential to ensure procedure durability.
